# Genetics and epidemiology of hypothyroidism and symmetrical onychomadesis in the Gordon setter and the English setter

**DOI:** 10.1186/s40575-015-0025-6

**Published:** 2015-08-21

**Authors:** Martine Lund Ziener, Stina Dahlgren, Stein Istre Thoresen, Frode Lingaas

**Affiliations:** Department of Basic Sciences and Aquatic Medicine, Norwegian University of Life Sciences, Oslo, Norway; Fredrikstad Animal Hospital, Fredrikstad, Norway

## Abstract

**Background:**

Hypothyroidism is one of the most common endocrine disorders, whereas symmetrical onychomadesis is a rare claw disease in the general dog population. The aims of this study were to estimate the prevalence of hypothyroidism and symmetrical onychomadesis in a birth cohort of 291 Gordon setters at eight years of age. Further, to describe the age at diagnosis of hypothyroidism in the 68 Gordon setters and 51 English setters included in the DLA study. Finally, to elucidate potential associations between dog leukocyte antigen (DLA) class II and hypothyroidism and/or symmetrical onychomadesis in the Gordon setter and the English setter.

**Results:**

In the birth cohort of eight years old Gordon setters, 2.7 % had hypothyroidism and 8.9 % had symmetrical onychomadesis, but only one out of these 291 dogs (0.3 %) had both diseases. Mean age at diagnosis of hypothyroidism for dogs included in the DLA study was 6.4 years (95 % CI: 5.6-7.2 years) in the Gordon setters and 7.7 years (95 % CI: 7.2-8.2 years) in the English setters. The DLA alleles most associated with hypothyroidism in the Gordon setter and English setter were DLA-DQB1*00201 (OR = 3.6, 95 % CI: 2.1-6.4, *p* < 0.001) and DLA-DQA1*00101 (OR = 2.9, 95 % CI: 1.3-6.6, *p* < 0.001), respectively.

In the Gordon setter, the haplotype DLA-DRB1*01801/DQA1*00101/DQB1*00802 was significantly associated with both symmetrical onychomadesis (OR = 2.9, 95 % CI: 1.7-5.2, *p* < 0.001) and with protection against hypothyroidism (OR = 0.3, 95 % CI: 0.2-0.5, *p* < 0.001).

**Conclusion:**

Hypothyroidism is a complex disease where DLA genes together with other genes may be involved in the pathogenesis of the disease. In the Gordon setter, one DLA haplotype that was associated with protection against hypothyroidism was also associated with symmetrical onychomadesis. These findings indicate that closely linked genes, instead of or together with the DLA genes themselves, may be associated with hypothyroidism and symmetrical onychomadesis. In a breed where several autoimmune diseases are prevalent all possible associations between DLA genes and actual diseases need to be investigated before DLA is considered used as a tool for marker-assisted selection.

**Electronic supplementary material:**

The online version of this article (doi:10.1186/s40575-015-0025-6) contains supplementary material, which is available to authorized users.

## Lay summary

An autoimmune disease occurs when the body’s immune system attacks normal cells of the body. For instance an autoimmune attack on the thyroid gland may cause hypothyroidism in both dogs and humans. Thyroid hormones are involved in several energy demanding processes in the body and subnormal hormone levels in dogs may cause a variety of clinical signs such as dullness, weight gain, lethargy, and skin changes. Dogs with compatible signs of hypothyroidism are commonly diagnosed based on subnormal levels of thyroid hormones and elevated levels of thyroid stimulating hormone (TSH) in serum.

Symmetrical onychomadesis is an acute claw disease where the dogs lose all claws during 3–4 months. The disease is known to occur in Gordon setters and many other dog breeds. Hypothyroidism and symmetrical onychomadesis appear to be more common in some dog breeds than others like the Rhodesian Ridgeback and the Giant Schnauzer. High prevalence of one disease in one dog breed could indicate that genetic factors contribute to disease development. In a birth cohort of Norwegian Gordon setters at eight years, the prevalence of hypothyroidism was 2.7 % and the prevalence of symmetrical onychomadesis was 8.9 % (*n* = 291). The Gordon setters and the English setters were middle aged when they were diagnosed with hypothyroidism.

Dog leukocyte antigen (DLA) genes play an essential role in the immune response and they have been associated with different autoimmune diseases in dogs. In this study, different DLA haplotypes and alleles were associated with hypothyroidism in the Gordon setter and the English setter. In the Gordon setter, one DLA haplotype that may protect against hypothyroidism may concurrently predispose dogs to symmetrical onychomadesis. All possible immune mediated diseases in one breed needs to be investigated for associations to DLA before information about DLA is considered used as a tool for marker-assisted selection to reduce disease prevalence.

## Background

Hypothyroidism is one of the most common endocrine diseases reported in dogs [1]. In most cases, the disease is considered to be the result of an autoimmune attack of the thyroid gland [2]. It is still debated whether the lymphocytic form of hypothyroidism is the first stage and the atrophic form is the end stage of the disease, or if they are two different diseases entities [2–4]. Subclinical hypothyroidism is characterised by the presence of autoantibodies against thyroglobulin (TgAA) in serum and elevated serum TSH values [2–4] with lymphocytic infiltrates in the thyroid gland [4]. In dogs with overt hypothyroidism, more than 75 % of the functional gland is destroyed and blood chemistry is characterised by the presence of TgAA, elevated TSH, and in most cases decreased free thyroxin/total thyroxin (FT4/TT4) levels [2–4]. End stage or atrophic form of hypothyroidism is characterised by absence of TgAA in serum, normal or elevated TSH, and decreased FT4/TT4 levels [2, 3]. The gland now has a non- inflammatory and an atrophic appearance on histopathology [4]. Hypothyroidism is considered to be a disease of middle aged dogs, but age at diagnosis has been reported to differ between breeds [4]. The age at diagnosis may also depend on the stage of hypothyroidism hence dogs with TgAA-positive hypothyroidism are in general younger than dogs with TgAA-negative hypothyroidism [2, 3].

Diagnosing hypothyroidism in dogs can be a challenge due to confusing blood results and vague clinical signs [2]. TSH, as a diagnostic test for hypothyroidism, has high specificity but low sensitivity in dogs [5]. A high TSH value is therefore well suited to confirm a diagnosis of hypothyroidism, but a normal TSH value cannot exclude hypothyroidism [5, 6]. Autoantibodies may also falsely increase TT4 and FT4 levels in serum when TT4 and FT4 are measured by chemiluminescense [7]. Moreover, dogs suffering from other critical diseases or severe chronic illnesses may have low TT4 values without having hypothyroidism (euthyroid sick syndrome) [8, 9]. These diagnostic aspects are important to consider when collecting cases and controls for genetic studies of hypothyroidism.

Autoimmune hypothyroidism is a multifactorial disease in which many genetic and environmental factors are assumed to affect disease development and disease outcome [2]. Comparably higher prevalences of hypothyroidism in the English setter, the Giant Schnauzer, and the Hovawart indicate an accumulation of genetic variants in these breeds [10, 11]. Research on autoimmune hypothyroidism in human patients indicates that genetic factors contribute to as much as 70-80 % to the risk of developing the disease [12, 13]. The major histocompatibility complex (MHC) class II has been associated with various autoimmune diseases in both humans and dogs [14, 15]. In dogs, genes coding for this complex are named DLA. The DLA class II genes comprise three highly polymorphic and closely linked genes, DLA-DRB1, DLA-DQA1, and DLA-DQB1 [16]. In the Doberman Pinscher, the Rhodesian Ridgeback and the Giant Schnauzer associations between specific DLA class II haplotypes/alleles and hypothyroidism have been described [17–20]. In the Gordon setter and the Giant Schnauzer associations between specific DLA class II haplotypes/alleles and symmetrical onychomadesis have been reported [21].

Common clinical signs of hypothyroidism in dogs include weight gain, mental dullness, and lethargy [3]. Other signs of the disease are hypothermia and poor hair coat quality with skin changes, such as alopecia, hyperkeratosis, and seborrhea [3, 4]. In humans, detached nails from the nail bed (onycholysis) has been described in hypothyroid patients [22]. Claw changes in dogs with hypothyroidism are uncommon [23]. Symmetrical onychomadesis is a claw disease where the dogs lose all their claws within a few months [24]. Both symmetrical onychomadesis and autoimmune hypothyroidism occur with high prevalence in Giant Schnauzers, German Shepherds and Rhodesian Ridgebacks [21, 23]. Mueller et al. suggested that these diseases may share genetic risk factors since they both occur with high prevalence in the same breeds, although not necessarily in the same dog [24, 25].

The aims of this study were to: 1) Investigate the prevalence of hypothyroidism and symmetrical onychomadesis in a large birth cohort of Gordon setters; 2) Describe the age at diagnosis of hypothyroidism in the Gordon setter and English setter; 3) To investigate potential associations between DLA class II haplotypes/alleles and hypothyroidism and/or symmetrical onychomadesis in the Gordon setter and the English setter.

## Results

### Epidemiology of hypothyroidism and symmetrical onychomadesis

The prevalence of hypothyroidism in eight years old Gordon setters was 2.7 % (95 % CI: 1.3-5.1 %, *n* = 291). The prevalence of symmetrical onychomadesis in the same study population was 8.9 % (95 % CI: 6.0-12.6 %). Only one of these 291 dogs had both hypothyroidism and symmetrical onychomadesis.

From the DLA study the Gordon setters comprised 68 cases and 93 controls and the English setters comprised 83 cases and 53 controls. The age at diagnosis, median TSH-, TT4-, FT4- and cholesterol values at time of diagnosis in cases are presented in Table 1. The mean age of the control dogs together with their median TSH-, TT4-, FT4- and cholesterol values are also presented in Table 1. TgAA analysis was not performed in all cases because it was not available at the time of samples collection. Thus only, 31 out of 151 hypothyroid dogs were tested for TgAA and 114 out of 146 controls were tested for TgAA (Table 1). Five of the controls had positive TgAA titers but these dogs were still included as controls because none of them developed clinical or biochemical signs of hypothyroidism during their life time.

**Table 1:** Gordon setters and English setters in the DLA study

	Cases		Controls	
	GS *(n = 68)*	ES *(n = 83)*	GS *(n = 93)*	ES *(n = 53)*
Sex and age				
Male	22	42	57	33
Female	46	41	36	20
Age (y): mean (95 % CI)	6.4 (5.6-7.2)	7.7 (7.2-8.2)	9.3 (9.0-9.7)	8.8 (8.3-9.3)
Range age, years	1.8-12.9	2.7-15	8-14.4	8-16
Analysis blood samples				
TSH ug/L: median (range)	3.01 (0.46-12.0)	2.24 (0.46-12.0)	0.14 (0.03-0.45)	0.23 (0.04-0.45)
TT4 nmol/L:	7.5 (4.0-77.0)	6.0 (4.0-44.0)	28.0 (16.0-53.0)	24.0 (16.0-45)
median (range)				
FT4 pmol/L:	9.0 (4.0-65.0)	7.0 (4.0-77.0)	14.0 (7.0-69.0)	14.0 (7.0-47.0)
median (range)				
Cholesterol mmol/L:	9.1 (2.6-20.8)	11.7 (3.4-23.1)	6.1 (3.6-11.0)	5.8 (3.8-11.0)
median (range)				
TgAA positive *(n)*	14	6	4	1
TgAA negative *(n)*	2	8	59	45
TgAA inconclusive *(n)*	0	1	0	5
Diagnosis of SO				
positive *(n)*	12	3	20	1
negative *(n)*	45	50	70	46

*TSH* thyroid stimulating hormone (reference values: 0–0.45 ug/L), *TT4* total thyroxin (Reference values: 16–46 nmol/L)*, FT4* free thyroxine (reference values: 7–44 pmol/L). cholesterol: (reference values: 3.4-10.0 mmol/L). *TgAA* thyroglobulin autoantibodies

Medical records of symmetrical onychomadesis were obtained in retrospect from 83.2 % of the Gordon setters and English setters participating in the DLA study (*n* = 297). Among the hypothyroid Gordon setters 21.1 % of these dogs had symmetrical onychomadesis (*n* = 57) and 5.6 % of the hypothyroid English setters had symmetrical onychomadesis (*n* = 53). In Gordon setters without hypothyroidism, 22.2 % of these dogs had symmetrical onychomadesis (*n* = 91) and 2.1 % of the English setters without hypothyroidism had symmetrical onychomadesis (*n* = 47) (Table 1).

### DLA haplotypes and alleles associated with hypothyroidism in the Gordon setter and the English setter

Ten DLA haplotypes were present in the Gordon setter population (*n* = 161), and seven DLA haplotypes were present in the English setter population (*n* = 136). Five DLA haplotypes occurred in both breeds (Tables 2 and 3). A complete overview of DLA allele frequencies in the Gordon setters and the English setters are given in Additional file 1: Tables S1and S2. The haplotype, DLA- DRB1*00103/DQA1*00101/DQB1*00201 was present in 8.3 % of the Gordon setters and found to be associated with hypothyroidism in this breed (OR = 4.4, 95 % CI: 1.8-11.5, *p* < 0.001) (Table 4). Only five Gordon setters were homozygous for this haplotype, three cases and two controls. The more common haplotype among the Gordon setters, DLA-DRB1*04901/DQA1*01001/DQB1*01901 was also associated with hypothyroidism (OR = 2.1, 95 % CI: 1.2-3.7, *p* = 0.008) (Table 4).

**Table 2:** DLA haplotypes in the Gordon setter

	All GS		GS cases		GS controls		GS cases/ GS controls		
DLA-haplotype	%	*n* = 322	%	*n* = 136	%	*n* = 186	OR	CI 95 %	*p*-value
DRB1*01801/DQA1*00101/DQB1*00802	31.9	103	18.3	25	41.9	78	0.3	0.2-0.5	0.000006
DRB1*01501/DQA1*00601/DQB1*02301	5.9	19	2.2	3	8.6	16	0.2	0.1-0.8	0.001
DRB1*00103/DQA1*00101/DQB1*00201	8.3	27	14.7	20	3.7	7	4.4	1.8-11.5	0.0005
DRB1*00101/DQA1*00101/DQB1*00201	11.1	36	15.4	21	8.0	15	2.1	1.03-4.28	0.04
DRB1*02001/DQA1*00401/DQB1*01303	11.4	37	11.0	15	11.8	22	n.s		
DRB1*04901/DQA1*01001/DQB1*01901	18.9	61	25.7	35	13.9	26	2.1	1.2-3.7	0.008
DRB1*00901/DQA1*00101/DQB1*008011	7.4	24	6.6	9	8.0	15	n.s		
DRB1*00501/DQA1*00301/DQB1*00501	0.9	3	1.4	2	0.5	1	n.s		
DRB1*00107/DQA1*00101/DQB1*00201	1.8	6	3.6	5	0.5	1	n.s		
DRB1*01501/DQA1*00601/DQB1*00301	1.8	6	0.7	1	2.6	5	n.s		

Cases; Gordon setter with hypothyroidism, Controls; Gordon setters without hypothyroidism, n.s; non significant, OR; Odd ratio, CI; confidence interval

**Table 3:** DLA haplotype in the English setter

	*All ES*		*ES cases*		*ES controls*		*ES cases/ES controls*		
DLA-haplotype	%	n = 272	%	n = 166	%	n = 106	OR	CI 95 %	*p*-value
DRB1*01501/DQA1*00601/DQB1*02301	2.1	5	0.6	1	3.8	4	n.s		
DRB1*00101/DQA1*00101/DQB1*00201	52.9	144	52.4	87	53.8	57	n.s		
DRB1*02001/DQA1*00401/DQB1*01303	4.0	11	3.6	6	4.7	5	n.s		
DRB1*00901/DQA1*00101/DQB1*008011	11.7	32	11.4	19	12.3	13	n.s		
DRB1*00601/DQA1*005011/DQB1*00701	3.3	9	1.2	2	6.6	7	0.2	0.03-0.8	0.02
DRB1*00107/DQA1*00101/DQB1*00201	24.6	67	29.5	49	17.0	18	2.0	1.1-3.8	0.01
DRB1*03802/DQA1*00901/DQB1*00101	1.4	4	1.3	2	1.8	2	n.s		

Cases; English setters with hypothyroidism, Controls; English setters without hypothyroidism, n.s; non significant, OR; Odd ratio, CI; confidence interval

**Table 4:** DLA-haplotypes and alleles associated with hypothyroidism or protection against hypothyroidism in the Gordon setter

	Total		Cases		Controls		Cases/controls		
DLA-haplotype or allele	%	*n* = 322	%	*n* = 136	%	*n* = 186	OR	95 % CI	*p*-value
DRB1*00103/DQA1*00101/DQB1*00201	8.3	27	14.7	20	3.7	7	4.4	1.8 - 11.5	0.0005
DLA-DQB1*00201	21.4	69	33.8	46	12.4	23	3.6	2.1 - 6.4	0.000005
DRB1*04901/DQA1*01001/DQB1*01901	18.9	61	25.7	35	13.9	26	2.1	1.2 - 3.7	0.008
DRB1*00101/DQA1*00101/DQB1*00201	11.1	36	15.4	21	8.0	15	2.1	1.0 - 4.3	0.04
DRB1*01801/DQA1*00101/DQB1*00802	31.9	103	18.3	25	41.9	78	0.3	0.2 - 0.5	0.000006
DRB1*01501/DQA1*00601/DQB1*02301	5.9	19	2.2	3	8.6	16	0.2	0.1 - 0.8	0.001

In the English setter the haplotype DLA-DRB1*00107/DQA1*00101/DQB1*00201 was associated with hypothyroidism (OR = 2.0, 95 % CI: 1.1-3.8, *p* = 0.01) (Table 5). Seven cases were homozygous, whereas none of the controls were homozygous for this haplotype. The allele DLA-DQA1*00101 was present in 89.3 % of the English setters and was found to be associated with hypothyroidism (OR = 2.9, 95 % CI: 1.3-6.6, *p* = 0.002) (Table 5).

**Table 5:** DLA-haplotypes and alleles associated with hypothyroidism or protection against hypothyroidism in the English setter

	Total		Cases		Controls		Cases/Controls		
DLA-haplotype or allele	%	*n* = 272	%	*n* = 166	%	*n* = 106	OR	CI 95 %	*p*-value
DRB1*00107/DQA1*00101/DQB1*00201	24.6	67	29.5	49	17.0	18	2.0	1.1-3.8	0.01
DLA-DQA1*00101	89.3	243	93.4	155	83.0	88	2.9	1.3-6.6	0.002
DLA-DQB1*00201	77.6	211	81.9	136	70.8	75	1.9	1.1-3.3	0.03
DRB1*00601/DQA1*005011/DQB1*00701	3.3	9	1.2	2	6.6	7	0.2	0.03-0.8	0.02

DLA- DQA1*00101 was also the most common allele in the Gordon setter, however, it was not associated with hypothyroidism in this breed. In the Gordon setter this allele was instead part of the most common and also protective haplotype DLA-DRB1*01801/DQA1*00101/DQB1*00802 (OR = 0.3, 95 % CI: 0.2-0.5, *p* < 0.001) (Table 4). Notably, DLA- DQB1*00201 was associated with hypothyroidism in both the Gordon setter (OR = 3.6, 95 % CI: 2.1-6.4, *p* < 0.001) and the English setter (OR = 1.9, 95 % CI: 1.1-3.3, *p* = 0.03). It is also worth noting that 77.6 % of the English setters and 21.4 % of the Gordon setters carried the allele DLA- DQB1*00201 (Tables 4 and 5).

Three Gordon setter cases and 19 controls were homozygous for the most common DLA haplotype in this breed (DLA-DRB1*01801/DQA1*00101/DQB1*00802). Thus, homozygosity for this DLA haplotype was associated with the strongest protection for hypothyroidism in the Gordon setter (OR = 0.2, 95 % CI: 0.1-0.6, *p* = 0.004). Eighteen Gordon setter cases and 41 controls were heterozygous for this haplotype (DLA-DRB1*01801/DQA1*00101/DQB1*00802) (OR = 0.51, 95 % CI: 0.29-0.98, *p* = 0.04).

Haplotype DLA-DRB1*01801/DQA1*00101/DQB1*00802 was absent in the English setter. The only DLA haplotype associated with protection for hypothyroidism in the English setter was DLA-DRB1*00601/DQA1*005011/DQB1*00701 (OR = 0.22, 95 % CI: 0.03-0.81, *p* = 0.02). Only nine English setters (3.3 %) and none of the Gordon setters had this haplotype. All alleles in the haplotype DLA-DRB1*00601/DQA1*005011/DQB1*00701 were unique to that haplotype.

### DLA haplotypes associated with symmetrical onychomadesis in the Gordon setter and the English setter

The haplotype DLA- DRB1*01801/DQA1*00101/DQB1*00802 has previously been associated with symmetrical onychomadesis in the Gordon setter [21] which was confirmed in the present study (Additional file 1: Table S3). Nine out of 12 Gordon setters with symmetrical onychomadesis and hypothyroidism were also heterozygous for this haplotype (DLA- DRB1*01801/DQA1*00101/DQB1*00802). Only three English setters had both symmetrical onychomadesis and hypothyroidism; one was homozygous for DLA- DRB1*00107/DQA100101/DQB1*00201, one was homozygous for DLA-DRB1*00101/DQA1*00101/DQB1*00201 and one was heterozygous with the preceding haplotypes.

## Discussion

Gordon setters and English setters are frequently used as hunting dogs and both hypothyroidism and symmetrical onychomadesis reduce their hunting abilities besides welfare concerns [26]. The true breed prevalence of hypothyroidism is challenging to determine. In this study, a large sample from a birth cohort of Gordon setters was investigated at eight years of age. The prevalence of hypothyroidism was 2.7 %. This estimate was based on dogs that were examined by a veterinarian because of their owners’ suspicion of hypothyroidism. The prevalence may therefore be underestimated because some owners misinterpret the signs of hypothyroidism as normal signs of getting old and hence do not seek medical care. The estimated prevalence of 8.9 % of Gordon setters affected with symmetrical onychomadesis is in accordance with previous published results [26]. The prevalence for hypothyroidism and symmetrical onychomadesis in the English setters could not be estimated in the present study, but this breed has been described as a high- risk breed for hypothyroidism in previous studies [3, 4]. Only four of the English setters in this study had symmetrical onychomadesis. The prevalence of symmetrical onychomadesis in the English setter is thought to be lower than that observed in the Gordon setter [26].

Overall, 50 % of hypothyroid dogs have TgAA positive hypothyroidism, but the frequencies of TgAA positive hypothyroidism vary among breeds [3]. This could suggest that different genetic factors affect disease development in various breeds [2, 3]. Graham et al. discovered that 61 out of 73 English setters (84 %) had TgAA positive hypothyroidism, while for instance only 13 out of 81 Dachshunds (16 %) were TgAA positive [3]. Graham et al. also discovered that dogs diagnosed with TgAA positive hypothyroidism in general were younger than dogs diagnosed with TgAA negative hypothyroidism [3]. In our study, English setters were relatively old when they were diagnosed with hypothyroidism, but since neither TgAA measurements nor biopsies of the thyroid gland were available for all the dogs in this present study, it cannot be determined if they had the atrophic form of hypothyroidism or a late form of TgAA-positive hypothyroidism.

DLA has been associated with several different immune mediated diseases in dogs [27–29]. The most common DLA haplotype in the Gordon setter (DLA-DRB1*01801/DQA1*00101/DQB1*00802) in this study was associated with protection for hypothyroidism, and interestingly, this DLA haplotype was also reported associated with symmetrical onychomadesis in the same breed. The fact that the same DLA haplotype is associated with one autoimmune disease and also associated with protection for another presumed autoimmune disease, clearly suggests that specific combinations of DLA alleles do not necessarily predispose dogs for autoimmune diseases in general. It also indicates that potential functional effects of autoimmune disease may be associated to closely linked loci to DLA-haplotypes. The allele DLA-DQA1*00101 was both associated with hypothyroidism in the English setter and part of a haplotype associated with protection of hypothyroidism in the Gordon setter. Several hypothyroidism protective DLA haplotypes occurred at a high frequency in the Gordon setter, whereas such protective DLA haplotypes were rare in the English setter. Another DLA haplotype associated with protection of hypothyroidism (DLA-DRB1*01301/DQA1*00301/DQB1*00501) has been reported from the Giant Schnauzer [20], but this haplotype was not observed among our Gordon setters or English setters. One previously reported DLA haplotype associated with hypothyroidism in the Giant Schnauzer and the Doberman (DLA-DRB1*01201/DQA1*00101/DQB1*00201) [18, 20] contained two of the most common alleles associated with hypothyroidism in English setter (DLA-DQA1*00101, DQB1*00201). The DLA-DQB1*00201 was also associated with hypothyroidism in the Gordon setter. This allele together with DLA-DQA1*00101 has also previously been reported associated with symmetrical onychomadesis in the Gordon setter [21]. Kennedy et al. suggested that the DLA-DQA1*00101 allele is associated with hypothyroidism in many breeds [17]. The findings of different DLA associations with hypothyroidism in different breeds are consistent with previous findings of different HLA associations with hypothyroidism observed both within and between ethnic groups in humans [15].

## Conclusions

The Gordon setters had a prevalence of 2.7 % of hypothyroidism and 8.9 % of symmetrical onychomadesis. Both the Gordon setters and the English setters were middle aged when they were diagnosed with hypothyroidism. One allele (DLA- DQB1*00201) was associated with hypothyroidism in both the Gordon setter and the English setter. Another allele (DLA- DQA1*00101) was associated with hypothyroidism in the English setter, but not in the Gordon setter. The same haplotype (DLA-DRB1*01801/DQA1*00101/DQB1*00802) was associated both with symmetrical onychomadesis and with protection against hypothyroidism in the Gordon setter. At the moment the functional genes in hypothyroidism and symmetrical onychomadesis are not known. The opposite effects of some alleles/haplotypes on two different autoimmune diseases may indicate that closely linked genes to DLA genes are involved. Further studies are needed to investigate how DLA genes or other closely linked genes participate in the pathogenesis of hypothyroidism and symmetrical onychomadesis. It is also important to state that selection of breeding animals supported by DLA haplotypes/alleles could only be used if taking into account also potential associations to other autoimmune disorders in a dog breed as well as how it influence the genetic variation in that breed.

## Methods

### Epidemiology of hypothyroidism and symmetrical onychomadesis

Gordon setters eight years of age received a health survey in 2012 (*n* = 474). Non-responders were followed up by telephone interview. The response rate was 61 % (*n* = 291). Dogs with reported hypothyroidism and/or symmetrical onychomadesis were confirmed by an evaluation of veterinary records. All dogs with hypothyroidism (*n* = 8) fulfilled the diagnostic criteria: TSH >0.45 μg/L, TT4 ≦16 nmol/L, and FT4≦8 pmol/L. Symmetrical onychomadesis cases were dogs with one or multiple episodes of onychomadesis during their lifetime (n = 26).

Cases and controls for the DLA study were collected between 2006 and 2012. Cases were analysed for serum values of TSH, TT4, FT4, and cholesterol (Table 1). Cases were dogs with clinical signs of hypothyroidism with serum TSH > 0.45 μg/L and at least one of the following diagnostic criteria; TT4 < 16 nmol/L and/or TT4 > 46 nmol/L, FT4 < 7 pmol/ and/ or FT4 > 44 pmol/L and/or cholesterol > 10 mmol/L and/or positive TgAA analysis. Controls were dogs eight years or older without signs of hypothyroidism and the following serum levels: TSH ≦ 0.45 μg/L, TT4 > 15 nmol/L, FT4 > 6 pmol/L, and cholesterol < 11 mmol/L (Table 1). The reference values at the Central laboratory was; TSH = 0–0.45 ug/L, TT4 = 16–46 nmol/L, FT4 = 7–44 pmol/L, cholesterol: 3.4-10.0 mmol/L. All dogs were screened for relatedness and showed no indication of a closer relationship than the general population in these breeds.

### Blood analysis

Serum levels of TSH, TT4, and FT4 were analysed on an IMMULITE® 2000 XPi Immunoassay System (Siemens Healthcare Diagnostics, Siemens AG, Germany). In the present study, two chemiluminescent assays for measuring free T4 in canine serum were used. Initially, an assay primarily evaluated for analyzing free T4 in human sera was used (IMMULITE® 2000 Free T4). However, this assay has been evaluated for canine samples [30]. When IMMULITE® 2000 Veterinary Free T4 became available this assay was preferred and calibrated to use identical reference intervals as related to the previous method for healthy and hypothyroid dogs. Most of the serum samples were analysed within five days, but a few samples had been kept frozen at −20 °C before they were analysed. Storage of serum samples at −20 °C should not interfere with serum TT4 and FT4 levels [31]. Analysis of TgAA antibodies was not available at the Central Laboratory until March, 2009. The presence of TgAA was determined by an indirect enzyme immunoassay using microplates containing both thyroglobulin coated (Tg+) and non-specific binding (Tg-) strips (Oxford Laboratories, Inc., Oxford, MI, USA) and an microplate absorbance reader (Tecan Sunrise™, Tecan Group Ltd., Switzerland) reading the absorbance at 450 nm for each well. The values for the non-specific binding wells were subtracted from the corresponding positive and negative sera. Then the average of the non-specific binding wells was subtracted from the average of each sample. Each sample was divided by the corrected positive reference serum and multiplied by 100. Samples > 25 % of the positive reference serum are positive for TgAA, samples below 10 % are negative and samples from 10 to 25 % are inconclusive (“grey zone”) for TgAA.

### DLA class II genotyping

DLA-DRB1, DLA-DQA1, and DLA-DQB1 exon 2 DNA sequences were obtained from 161 Gordon Setters and 136 English Setters. DNA was extracted using E.Z.N.A® blood DNA kit and then PCR amplified. One PCR reaction mixture contained 1.5 μl aliquots of the DNA solution, 0.05 μl HotStarTaq Master Mix (Qiagen GmbH), 0.5 μl of each forward and reverse DLA-specific primer (20 pmol; Eurofins MWG Operon) (see Additional file 1: Table S4), 1.5 μl dNTP (2.5 mM; Amplicon), 1.5 μl PCR Buffer (Qiagen), 1 μl Q-Solution (Qiagen), and 8.45 μl distilled water. PCR reactions were carried out in a Veriti**®** Thermal Cycler Applied Biosystems using the following PCR protocol: initial Hot Start at 95 °C for 15 min; 14 touch down cycles of 95 °C for 30 s, followed by 1 min annealing, starting at 62 °C (DLA-DRB1), 54 °C (DLA-DQA1), 73 °C (DLA-DQB1) and reducing by 0.5 °C each cycle, and 72 °C for 1 min; then 20 cycles of 95 °C for 30 s, 55 °C (DLA-DRB1), 47 °C (DLA-DQA1) 66 °C (DLA-DQB1) for 1 min, 72 °C for 1 min; and final extension at 72 °C for 10 min. PCR products were separated on 1 % agarose gel and visualized under UV light after staining with ethidium bromide to check for appropriately sized products, and subsequently purified using the Illustra™ Exostar™ clean-up kit (GE Healthcare). Sequencing reaction mixtures were prepared using BigDye**®** Terminator v3.1 Cycle Sequencing Kit (Thermo Fisher Scientific) and the samples were then sequenced on a 3500xL Genetic Analyzer (Applied Biosystems). PCR products were initially sequenced in one direction, but if the DLA allele could not be completely determined the opposite direction was sequenced as well. All DNA sequences were finally analysed and alleles were designated for each sequence using MatchTools and MatchToolsNavigator (Applied Biosystems). Alleles not present in the built-in allele library were identified by a BLAST search [32].

### Statistical analyses

Standard deviations and confidence intervals for means were calculated using Microsoft Excel 2011. In hypothyroid dogs, the standard deviations were all greater than half of the mean for the TSH, TT4 and FT4 values. This strongly indicated that the thyroid values were skewed and therefore the median values with range were reported instead of the mean with standard deviation [33]. Two by two contingency tables were used to evaluate association between DLA-DRB1/DQA1/DQB1 haplotypes and individual DLA alleles. Odd ratio (OR) were calculated using the web-based statistical calculator in OpenEpi 3.01. Chi-square test was used and two-tail mid-p exact values were calculated. A 95 % confidence level was used for all tests.

### Ethics statement

Certified veterinarians collected the blood samples during routine clinical consultations, and in agreement with the provisions enforced by the Norwegian Animal Research Authority. Ethical approval for collecting blood samples is not required in Norway (Regulation on Animal Experimentation of January 15, 1996, in accordance with the Animal Welfare Act of June 19, 2009). For each of the dogs that participated in the study, the consent of its owner was obtained prior to inclusion.

## Additional file

Additional file 1: Table S1. DLA alleles in the Gordon setter (GS). **Table S2.** DLA alleles in the English setter (ES). **Table S3.** DLA haplotypes associated with symmetrical onychomadesis and protection for symmetrical onychomadesis in the Gordon setter. **Table S4.** Oligonucleotide primers used in the study. (DOCX 26 kb)

## Abbreviations

DLA: Dog leukocyte antigen
FT4: Free thyroxin
HLA: Human leukocyte antigen
LD: Linkage disequilibrium
MHC: Major histocompatibility complex
OR: Odds ratio
TgAA: Thyroid globulin autoantibodies
TSH: Thyroid stimulating hormone
TT4: Total thyroxin
