# Supplementary material for: Genetics and epidemiology of hypothyroidism and symmetrical onychomadesis in the Gordon setter and the English setter
Source: Canine Genet Epidemiol. 2015 Aug 21;2:12. doi: 10.1186/s40575-015-0025-6 (PMC4579390; doi:10.1186/s40575-015-0025-6)
Supplement: Additional file 1: Table S1. — DLA alleles in the Gordon setter (GS). Table S2. DLA alleles in the English setter (ES). Table S3. DLA haplotypes associated with symmetrical onychomadesis and protection for symmetrical onychomadesis in the Gordon setter. Table S4. Oligonucleotide primers used in the study. (DOCX 26 kb) [file 40575_2015_25_MOESM1_ESM.docx]

Additional file 1

| **Table S1 DLA alleles in the Gordon setter (GS)** | | | | | | | | | |
| --- | --- | --- | --- | --- | --- | --- | --- | --- | --- |
|  |  |  |  |  |  |  |  | | |
| Allele | All GS | All GS | GS cases | GS cases | GS controls | GS controls | GS cases/GS controls | | |
| DLA-DRB1 | % | n=322 | % | n=136 | % | n=186 | OR | CI 95% | p-value |
| 01801 | 32.0 | 103 | 18.4 | 25 | 41.9 | 78 | 0.3 | 0.2-0.5 | 0.000006 |
| 01501 | 7.8 | 25 | 2.9 | 4 | 11.3 | 21 | 0.2 | 0.1-0.8 | 0.001 |
| 00103 | 8.4 | 27 | 14.0 | 20 | 3.8 | 7 | 4.4 | 1.8-11.5 | 0.0005 |
| 00101 | 11.2 | 36 | 15.4 | 21 | 8.1 | 15 | 2.0 | 1.03-4.28 | 0.04 |
| 02001 | 11.5 | 37 | 11.0 | 15 | 11.8 | 22 | n.s |  |  |
| 04901 | 18.9 | 61 | 25.7 | 35 | 14.0 | 26 | 2.1 | 1.2-3.7 | 0.004 |
| 00901 | 7.5 | 24 | 6.6 | 9 | 8.1 | 15 | n.s |  |  |
| 00501 | 0.9 | 3 | 1.5 | 2 | 0.5 | 1 | n.s |  |  |
| 00107 | 1.9 | 6 | 3.7 | 5 | 0.5 | 1 | n.s |  |  |
| DLA-DQA1 |  |  |  |  |  |  |  |  |  |
| 00101 | 60.9 | 196 | 58.8 | 80 | 62.4 | 116 | n.s |  |  |
| 00601 | 7.8 | 25 | 2.9 | 4 | 11.3 | 21 | 0.2 | 0.1-0.8 | 0.001 |
| 00401 | 11.5 | 37 | 11.0 | 15 | 11.8 | 22 | n.s |  |  |
| 01001 | 18.9 | 61 | 25.7 | 35 | 14.0 | 26 | 2.1 | 1.2-3.7 | 0.004 |
| 00301 | 0.9 | 3 | 1.5 | 2 | 0.5 | 1 | n.s |  |  |
| DLA-DQB1 |  |  |  |  |  |  |  |  |  |
| 00802 | 32.0 | 103 | 18.4 | 25 | 41.9 | 78 | 0.3 | 0.2-0.5 | 0.000006 |
| 02301 | 5.9 | 19 | 2.2 | 3 | 8.6 | 16 | 0.2 | 0.1-0.8 | 0.001 |
| 00201 | 21.4 | 69 | 33.8 | 46 | 12.4 | 23 | 3.6 | 2.1-6.4 | 0.000005 |
| 01303 | 11.5 | 37 | 11.0 | 15 | 11.8 | 22 | n.s |  |  |
| 01901 | 18.9 | 61 | 25.7 | 35 | 14.0 | 26 | 2.1 | 1.2-3.7 | 0.004 |
| 008011 | 7.5 | 24 | 6.6 | 9 | 8.1 | 15 | n.s |  |  |
| 00501 | 0.9 | 3 | 1.5 | 2 | 0.5 | 1 | n.s |  |  |
| 00301 | 1.9 | 6 | 0.7 | 1 | 2.7 | 5 | n.s |  |  |

| **Table S2 DLA alleles in the English setter (ES)** | | | | | | | | | |
| --- | --- | --- | --- | --- | --- | --- | --- | --- | --- |
| Allele | All ES | All ES | ES cases | ES cases | ES controls | ES controls | ES cases/ ES controls | | |
| DLA-DRB1 | % | n=27 | % | n=166 | % | n=106 | OR | CI 95% | P value |
| 01501 | 1.8 | 5 | 0.6 | 1 | 3.8 | 4 | n.s |  |  |
| 00101 | 53.1 | 144 | 52.4 | 87 | 53.8 | 57 | n.s |  |  |
| 02001 | 4.1 | 11 | 3.6 | 5 | 4.7 | 6 | n.s |  |  |
| 00901 | 11.8 | 32 | 11.4 | 19 | 12.3 | 13 | n.s |  |  |
| 00601 | 3.3 |  | 1.2 | 2 | 6.6 | 7 | 0.2 | 0.03-0.8 | 0.02 |
| 00107 | 24.7 | 67 | 29.5 | 49 | 17.0 | 18 | 2.0 | 1.1-3.8 | 0.01 |
| 03802 | 1.5 | 4 | 1.2 | 2 | 1.9 | 2 | n.s |  |  |
| DLA-DQA1 |  |  |  |  |  |  |  |  |  |
| 00601 | 1.8 | 5 | 0.6 | 1 | 3.8 | 4 | n.s |  |  |
| 00101 | 89.3 | 243 | 93.4 | 155 | 83.0 | 88 | 2.9 | 1.3-6.6 | 0.002 |
| 00401 | 4.0 | 11 | 3.6 | 6 | 4.7 | 5 | n.s |  |  |
| 005011 | 3.3 | 9 | 1.2 | 2 | 6.6 | 7 | 0.2 | 0.03-0.8 | 0.02 |
| 00901 | 1.5 | 4 | 1.2 | 2 | 1.9 | 2 | n.s |  |  |
| DLA-DQB1 |  |  |  |  |  |  |  |  |  |
| 02301 | 1.8 | 5 | 0.6 | 1 | 3.8 | 4 | n.s |  |  |
| 00201 | 77.6 | 211 | 81.9 | 136 | 70.8 | 75 | 1.9 | 1.05-3.3 | 0.03 |
| 01303 | 4.0 | 11 | 3.6 | 6 | 4.7 | 5 | n.s |  |  |
| 008011 | 11.8 | 32 | 11.4 | 19 | 12.3 | 13 | n.s |  |  |
| 00701 | 3.3 | 9 | 1.2 | 2 | 6.6 | 7 | 0.2 | 0.03-0.8 | 0.02 |
| 00101 | 1.5 | 4 | 1.2 | 2 | 1.9 | 2 | n.s |  |  |
| Cases; English setter with hypothyroidism, Controls; English setter without hypothyroidism, CI; confidence interval, OR; odds ratio, n.s; non significant | | | | | | | | | |

| **Table S3 DLA haplotypes associated with symmetrical onychomadesis and protection for symmetrical onychomadesis in the Gordon setter** | | | | | | | | | |
| --- | --- | --- | --- | --- | --- | --- | --- | --- | --- |
|  | GS total | | GS cases | | GS controls | | GS cases/ GS controls | | |
| DLA-haplotype | % | n=294 | % | n=64 | % | n=230 | OR | 95 % CI | p-value |
| DRB1*01801/DQA1*00101/DQB1*00802 | 32,0 | 94 | 51,6 | 33 | 26,5 | 61 | 2.9 | 1.7-5.2 | 0.0002 |
| DRB1*01501/DQA1*00601/DQB1*02301 | 2,0 | 6 | 0,0 | 0 | 2,6 | 6 | n.s |  |  |
| DRB1*00103/DQA1*00101/DQB1*00201 | 8,2 | 24 | 7,8 | 5 | 8,3 | 19 | n.s |  |  |
| DRB1*00101/DQA1*00101/DQB1*00201 | 10,5 | 31 | 12,5 | 8 | 10,0 | 23 | n.s |  |  |
| DRB1*02001/DQA1*00401/DQB1*01303 | 11,6 | 34 | 7,8 | 5 | 12,6 | 29 | n.s |  |  |
| DRB1*04901/DQA1*01001/DQB1*01901 | 18,7 | 55 | 12,5 | 8 | 20,4 | 47 | n.s |  |  |
| DRB1*00901/DQA1*00101/DQB1*008011 | 7,8 | 23 | 3,1 | 2 | 9,1 | 21 | n.s |  |  |
| DRB1*00501/DQA1*00301/DQB1*00501 | 1,0 | 3 | 0,0 | 0 | 1,3 | 3 | n.s |  |  |
| DRB1*00107/DQA1*00101/DQB1*00201 | 1,7 | 5 | 1,6 | 1 | 1,7 | 4 | n.s |  |  |
| DRB1*01501/DQA1*00601/DQB1*00301 | 6,5 | 19 | 3,1 | 2 | 7,4 | 17 | n.s |  |  |
| Cases; Gordon setter with symmetrical onychomadesis, Controls; Gordon setter without symmetrical onychomadesis, CI; confidence interval, OR; odds ratio, n.s; non significant | | | | | | | | | |

**Table S4 Oligonucleotide primers used in the study**

| **Primer** | **Primer sequence** |
| --- | --- |
| DLA-DRB1 (forward) | 5’-CCGTCCCCACAGCACATTTC-3’ |
| DLA-DRB1 (reverse)^1^ | 5’- TAATACGACTCACTATAGGGTGTGCTACACACCTCAGCACCA-3’ |
| DLA-DQA1 (forward) | 5’-CTCTCACGGAGCATACACGA-3’ |
| DLA-DQA1 (reverse) | 5’-ATGCTAGGGAGGAAGGGAAA-3’ |
| DLA-DQB1 (forward)^1^ | 5’-TAATACGACTCACTATAGGGCTCACTGGCCCGGCTGTCTC-3’ |
| DLA-DQB1 (reverse) | 5’-CACCTCGCCGCTGCAACGTG-3’ |
| T7 | 5’-TAATACGACTCACTATAGGG-3’ |

^1^Primer tailed with T7 promoter at the 5' end.
